# Blood Pressure Modifies Retinal Susceptibility to Intraocular Pressure Elevation

**DOI:** 10.1371/journal.pone.0031104

**Published:** 2012-02-16

**Authors:** Zheng He, Christine T. O. Nguyen, James A. Armitage, Algis J. Vingrys, Bang V. Bui

**Affiliations:** 1 Department of Optometry and Vision Sciences, University of Melbourne, Parkville, Victoria, Australia; 2 Department of Anatomy and Developmental Biology, Monash University, Clayton, Victoria, Australia; Massachusetts Eye & Ear Infirmary, Harvard Medical School, United States of America

## Abstract

Primary open angle glaucoma affects more than 67 million people. Elevated intraocular pressure (IOP) is a risk factor for glaucoma and may reduce nutrient availability by decreasing ocular perfusion pressure (OPP). An interaction between arterial blood pressure and IOP determines OPP; but the exact contribution that these factors have for retinal function is not fully understood. Here we sought to determine how acute modifications of arterial pressure will affect the susceptibility of neuronal function and blood flow to IOP challenge. Anaesthetized (ketamine:xylazine) Long-Evan rats with low (∼60 mmHg, sodium nitroprusside infusion), moderate (∼100 mmHg, saline), or high levels (∼160 mmHg, angiotensin II) of mean arterial pressure (MAP, n = 5–10 per group) were subjected to IOP challenge (10–120 mmHg, 5 mmHg steps every 3 minutes). Electroretinograms were measured at each IOP step to assess bipolar cell (b-wave) and inner retinal function (scotopic threshold response or STR). Ocular blood flow was measured using laser-Doppler flowmetry in groups with similar MAP level and the same IOP challenge protocol. Both b-wave and STR amplitudes decreased with IOP elevation. Retinal function was less susceptible to IOP challenge when MAP was high, whereas the converse was true for low MAP. Consistent with the effects on retinal function, higher IOP was needed to attenuated ocular blood flow in animals with higher MAP. The susceptibility of retinal function to IOP challenge can be ameliorated by acute high BP, and exacerbated by low BP. This is partially mediated by modifications in ocular blood flow.

## Introduction

Glaucoma is the second leading cause of blindness in those of working age and is characterized by a progressive death of the cells that make up the optic nerve [1]. The most well documented risk factor for glaucoma is elevated eye pressure (intraocular pressure, IOP). Elevated IOP is thought to cause retinal damage by mechanical and vascular mechanisms. The vascular compromise is thought to occur through direct compression of blood vessels in the optic nerve head and retina. When IOP elevation reduces ocular perfusion pressure (OPP) beyond the capacity for autoregulation, ocular blood flow will become compromised leading to retinal dysfunction. Since OPP represents a balance between mean arterial pressure and IOP (OPP = MAP−IOP), it is likely that a reduction in systemic blood pressure (BP) or a comparable increase in IOP will have similar effects on retinal function. Thus, for a given IOP elevation, retinal dysfunction should be exacerbated by low BP, but ameliorated by high BP.

Several studies suggest that blood pressure plays an important role in glaucoma. More specifically, nocturnal hypotension may exacerbate the progression of visual field loss in patients with glaucoma [2]–[4]. It is thought that when a nocturnal BP dip coincides with an IOP spike, a substantial OPP reduction produces an intermittent insult that increases the risk of disease progression [5]. In agreement with this hypothesis low BP has consistently been found to be a risk factor in glaucoma [6]–[15]. However, epidemiological studies offer equivocal conclusions as to whether systemic hypertension reduces glaucoma risk. A number of large scale epidemiological trials report a lower risk of glaucoma in individuals with elevated blood pressure [12], [15]–[17], whereas others [13], [18]–[21] have found the opposite. This suggests that the influence of systemic hypertension on glaucoma is complex [22]. On the one hand, one might expect high BP to improve OPP and provide protection against IOP elevation. On the other hand, systemic hypertension may be complicated by vascular dysfunction, which may counteract any protective effect afforded by high BP.

Given the above, a better understanding of how IOP and BP interact to influence retinal function is needed. An acute experimental approach that affords accurate simultaneous control of both IOP and BP is useful as it avoids long term cardiovascular confounds. Few studies have measured retinal function during simultaneous manipulation of IOP and BP [23], [24]. To date, no study has measured retinal function and blood flow during IOP and BP manipulation. Thus the aim of this study is to evaluate retinal function and blood flow over a wide range of OPPs, induced by IOP elevation in rats with low, moderate and high levels of acutely modified BP.

## Materials and Methods

### Subjects

All experimental procedures were in compliance with the ARVO Statement for the Use of Animals in Ophthalmic and Vision Research and the NHMRC Australian Code of Practice for the care and use of animals for scientific purposes. Animal ethics approval was obtained from the Animal Ethics Committee, Science Faculty, University of Melbourne. Animals used in this study were male Long-Evans rats (aged 10–15 weeks, 300–400 g, Monash Animal Service, Clayton, VIC, Aust). Rats were housed in a 12-hour light (50 lux) /12-hour dark environment with normal rat chow and water available *ad libitum*. All experimental procedures were conducted under general anaesthesia (ketamine∶xylazine, 60∶5 mg/kg, im). As ketamine is known to affect blood pressure in rodents [25], care was taken to standardise the anaesthesia regimen and duration in all animals. A topical anaesthetic (proxymetacaine 0.5% eye drops) and mydriatic (0.5% tropicamide) was given as necessary.

### Acute IOP elevation

**Figure 1:**
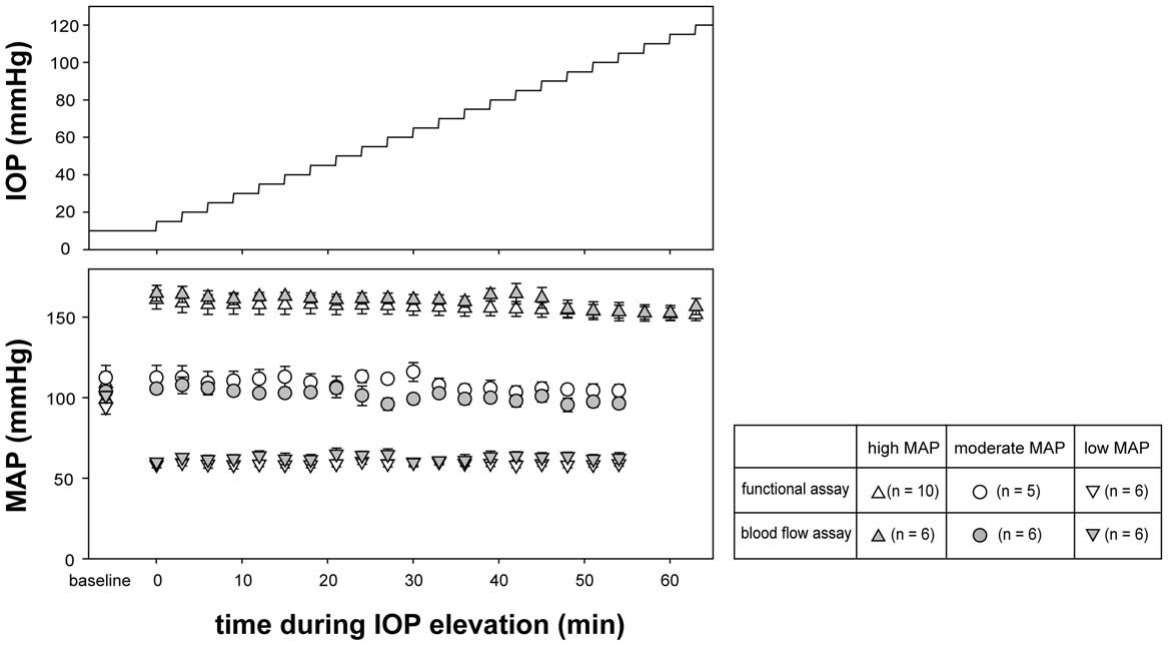
Blood pressure during experimental manipulations. Experimental protocol for stepwise IOP elevation in animals with acute high, moderate and low blood pressure. MAP (mean ± SEM) was held stable during IOP elevation. In each blood pressure group, animals were assigned to either functional (white symbols) or blood flow assay (grey symbols). MAP levels in animals in the functional assay were similar to their counterparts in the blood flow assay (p>0.05).

To assess ocular susceptibility across a gradient of OPPs, animals underwent stepwise IOP elevation from 10 to 120 mmHg (increment of 5 mmHg for 3 min) while MAP was held stable at low, moderate or high level (Figure 1). Each BP cohort was subdivided into two groups to measure either retinal function or ocular blood flow during IOP challenge. In the functional assay, IOP was raised manometrically by vitreal chamber cannulation, whereas in the blood flow assay, IOP was raised manometrically by anterior chamber cannulation. Vitreal chamber cannulation was chosen during functional assay to avoid interference with the placement of electroretinogram (ERG) electrodes on the cornea. Anterior chamber cannulation was used for ocular blood flow measurement to allow placement of a 26G laser-Doppler flowmetry (LDF) probe in the vitreal chamber. A pilot study showed that both vitreal and anterior chamber cannulations produce the same level of IOP elevation (Figure S1). Following cannulation, the desired IOP was achieved by placing a saline reservoir at a precalibrated height.

### Blood pressure monitoring and manipulation

Blood pressure was monitored via an indwelling cannula in the left femoral artery. Blood pressure was modulated by pharmacological intervention. Blood pressure reduction and elevation was achieved by variable infusion into the femoral vein of sodium nitroprusside (SNP, 84 mmol/ L in 0.9% saline, Sigma Aldrich, Castle Hill, Australia) and angiotensin II (AngII, 10 mmol/L in 0.9% saline, Auspep, Parkville, Australia), respectively.

To cannulate the femoral arteries and veins, the left groin area was shaved and disinfected with 70% ethanol. The left femoral artery and vein were exposed with a 2 cm skin incision and separated from the surrounding connective tissue by blunt dissection. A small incision was made through the arterial wall. A heparinised polyethylene cannula (inner and outer diameter 0.28 and 0.61 mm respectively) was inserted 3 cm proximally into the femoral artery. The exterior end of the cannula was connected to a pressure transducer (Transpac, Abbott Critical Care Systems, Sligo, IRE) to give direct and continuous BP monitoring.

Using the same method, the femoral vein was also cannulated, which allowed systemic BP to be maintained at low, moderate and high levels by infusion of SNP (2.5%; 50–250 µg/kg/min), normal saline (5 µl/min) or AngII (1%; 45–90 µg/kg/min), respectively. The rate of infusion was constantly adjusted within these ranges using an electronic syringe pump (HA22I, Harvard Apparatus, MA, USA) to produce a stable BP level during the simultaneous stepwise IOP elevation (Figure 1). The average MAP in the functional assay was 63±2, 100±3, and 161±4 mmHg (low, moderate and high BP groups respectively), which are similar to the groups used for the blood flow (59±2, 108±4 and 156±5 mmHg; p = 0.934, two-way ANOVA). As MAP was similar between animals for retinal function and ocular blood flow assays, the two outcome measures could be directly compared, despite being obtained from different cohorts of animals.

While one eye underwent IOP challenge, the fellow eye served as control with IOP set manometrically to 10 mmHg via vitreal chamber cannulation. Retinal function was found to remain unchanged in these sham control eyes (Figure S2). This suggests that, under current experimental conditions, SNP and AngII infusion do not affect retinal function.

In this study, OPP is calculated as the difference between mean arterial pressure and IOP (OPP = MAP−IOP), which differs slightly from the relationship used in humans (OPP = 2/3 MAP−IOP). The ‘2/3’ MAP adjustment factor is only applicable to primates in an upright position and therefore not incorporated here, as rat OPP is measured in a supine position. This estimate of OPP for rats is consistent with previous estimates from pigs [26], cats [27], [28] and rabbits [29].

### Retinal function: electroretinography

To quantify retinal susceptibility to IOP challenge, scotopic electroretinograms (ERG) were recorded at each IOP step. In rodents, the scotopic b-wave reflects rod bipolar cell function [30], and the scotopic threshold response (STR) is representative of retinal ganglion cells [31], [32]. The ganglion cell contribution to the STR has been established in previous studies of experimental glaucoma [33]–[35], optic nerve transection [31], as well as those using pharmacological agents [31], [32], [36] and behavioral approaches [37]. One limitation of the STR is that its signal-to-noise ratio is smaller than the b-wave. To address this issue, the STR recorded at each IOP step represents an average of 20 repeats.

Prior to functional measurement, animals were dark-adapted overnight (12 hours). To maintain dark-adaptation, all procedures including simultaneous acute IOP elevation and BP manipulations were performed in darkness with the aid of night vision goggles (NVMT24021, Yukon Advanced Optics®, USA) and an infra-red light source (L = 880 nm, 18 LED IR Spotlight). Anaesthetized rats were placed on a heated platform. The active electrode (custom-made chlorided silver electrode) was placed on the cornea, and the ring-shaped reference electrode positioned around the limbus. The ground electrode was inserted subcutaneously in the tail. At each IOP step, a pair of stimuli of −1.12 and −5.25 log cd.s.m^−2^ was presented via a Ganzfeld integrating sphere (Photometric Solutions International, VIC, Aust), which elicited the b-wave and the STR (STR averaged over 20 repeats), respectively. Signals were digitized at 4 kHz with ×1000 amplification and a band-pass of 0.3 to 1000 Hz (−3 dB). Both b-wave and STR were quantified in terms of the peak-to-trough amplitude.

### Ocular blood flow: laser-Doppler flowmetry

Laser-Doppler flowmetry (LDF; ML191, ADInstruments Pty Ltd, NSW, Australia) was employed to measure ocular blood flow by inserting an invasive needle probe (MNP110XP, ADInstruments) into the vitreal chamber. The working principle of LDF and its applications in the eye are detailed elsewhere [38]–[41]. Briefly, a Doppler frequency shift of the incident laser light is produced by moving blood cells but not by static tissue. Within a given measurement volume, this Doppler shift is processed to derive blood flow. Prior to IOP elevation, a small puncture was made at the 12 o'clock position, 1 mm posterior to the limbus using a 22G needle, which provides access for the LDF probe into the vitreal chamber. Using a micromanipulator (KITE, World Precision Instruments, FL, USA), the tip of the LDF probe was inserted 2 mm intravitreally pointing towards the retina. The measurement depth of the LDF probe is 1 mm [42], which in rats reflects a weighted average of both retinal and choroidal blood flow (Figure S3). Therefore, the term “ocular blood flow” is used throughout this study.

### Normalization of relative ocular blood flow

For any given eye, the LDF provides a reliable intra-subject comparison and is excellent for continuous blood flow monitoring, which makes it ideal for assessing a relative change in response to IOP challenge [43]. To allow for inter-subject comparison between blood pressure groups, baseline flow (IOP = 10 mmHg) in different groups was compared by characterizing the effect of MAP variation on ocular blood flow. A full MAP-flow curve was collected in a separate group of animals (n = 5) with IOP kept at 10 mmHg. More specifically, blood flow was measured over a wide range of MAPs (25–165 mmHg) by using a combination of renal and coeliac artery ligation to produce hypertension, followed by variable SNP infusion to produce hypotension. This curve was used to determine the baseline blood flow for each animal prior to IOP elevation. Blood flow measured during IOP challenge, was then normalized to each animals adjusted baseline.

### Data analysis

All group data were expressed as mean ± SEM. At baseline, ERG and LDF measurements were found to be normally distributed (Kolmogorov-Smirnov normality statistics) with equal variance. ERG amplitudes were also expressed relative to baseline (IOP of 10 mmHg). The relationship between IOP and relative retinal function (b-wave and STR amplitude) was described using a cumulative normal function [33],
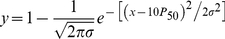
(1)where, relative retinal function (y, %) is described as a function of IOP (x, mmHg). The parameter σ indicates the steepness of the curve, whereas IOP_50_ represents the IOP for a 50% reduction in retinal function, which provides a measure of susceptibility to IOP elevation. To consider the association between blood pressure and function or blood flow susceptibility to IOP challenge, the correlation between MAP and IOP_50_ was examined with a Deming regression, which is a robust method of regression that accounts for the variability in both axes (MAP and IOP_50_). Equation 1 was also fitted to relative retinal function plotted against OPP, where OPP_50_ is the index of susceptibility.

## Results

### Influence of blood pressure on functional susceptibility to IOP

**Figure 2:**
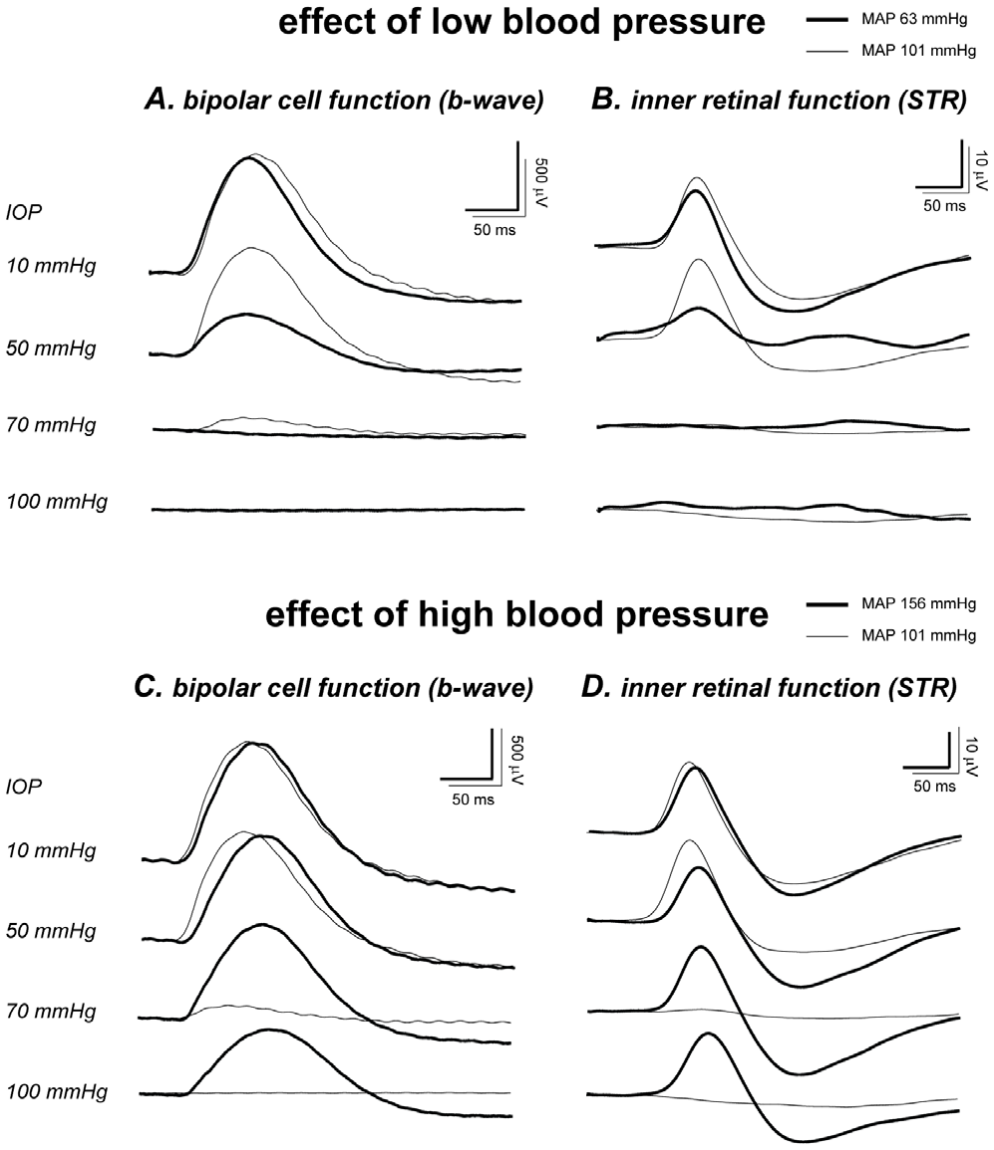
Effect of blood pressure on ERG waveforms during IOP elevation. Representative ERG b-wave and STR (to −1.12 and −5.25 log cd.s.m^−2^ stimuli, respectively) showing the effect of low BP (**A & C**, thick traces, MAP 63 mmHg) and high BP (**B & D**, thick traces, MAP 156 mmHg) on retinal susceptibility to IOP elevation. Overlaid with the hypo- and hypertensive responses is that from a control rat with moderate blood pressure (thin traces, MAP 101 mmHg).

Figure 2 shows individual examples of ERG b-wave (Panels A and C) and STR (Panels B and D) responses recorded during simultaneous BP and IOP manipulation. At baseline IOP, waveforms in the hypotensive rat (MAP 63 mmHg, thick traces, Panels A and B) were similar to those in the control (MAP 101 mmHg, thin traces) despite differences in BP. The effect of BP becomes manifest as IOP increases. In particular, the b-wave and STR were reduced to a greater extent in the hypotensive when compared with the normotensive rat.

The same control animal (moderate MAP, 101 mmHg) shown in Figures 2A and 2B (thin traces) was replotted in Figures 2C and 2D, to allow comparison with a hypertensive animal (MAP 156 mmHg, thick traces). Baseline b-wave and STR in the hypertensive rat were not different to the control. IOP elevation produces less dysfunction in the hypertensive rat. This was especially evident at 100 mmHg, where retinal function was abolished in the normotensive rat (MAP 101 mmHg, OPP∼0 mmHg) but preserved in the hypertensive animal (MAP 156 mmHg, OPP 56 mmHg).

**Figure 3 pone-0031104-g003:**
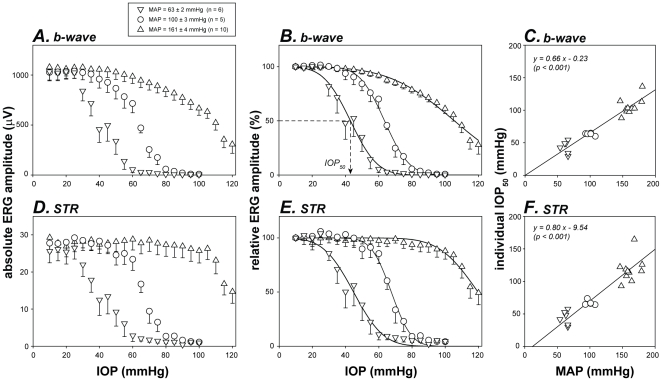
Effect of BP on the susceptibility of retinal function to IOP elevation. Given that baseline function was similar between low, moderate and high BP groups, the absolute amplitudes of b-wave (A) and STR (D) are also expressed relative (B & E) to their own baselines. Curves: cumulative normal function (Equation 1). Arrow: definition of IOP_50_ as the IOP for 50% ERG deficit. Error bars: SEM. Relationships between individual IOP_50_ and MAP for b-wave (**C**) and STR (**F**) are modelled using a Deming regression.

Figure 3 shows the group data (mean ± SEM) for absolute and relative ERG amplitude as a function of IOP. At baseline IOP (10 mmHg), absolute b-wave and STR amplitudes were similar between the three BP groups (p = 0.881 and 0.720 for Figures 3A and 3D, respectively; one-way ANOVA), indicating that neither changes in MAP (from 63 to 161 mmHg) nor the pharmacological agents *per se* (AngII and SNP) had any detectable effect on the ERG. Therefore b-wave and STR amplitude were normalized to their own baseline (Figures 3B and 3E). When comparing BP groups, the IOP-response curve gradually shifts rightward with increasing MAP (interaction p<0.001 for both Figures 3B and 3E, two-way RM ANOVA), meaning that retinal function was more resistant to IOP as BP increased.

**Figure 4:**
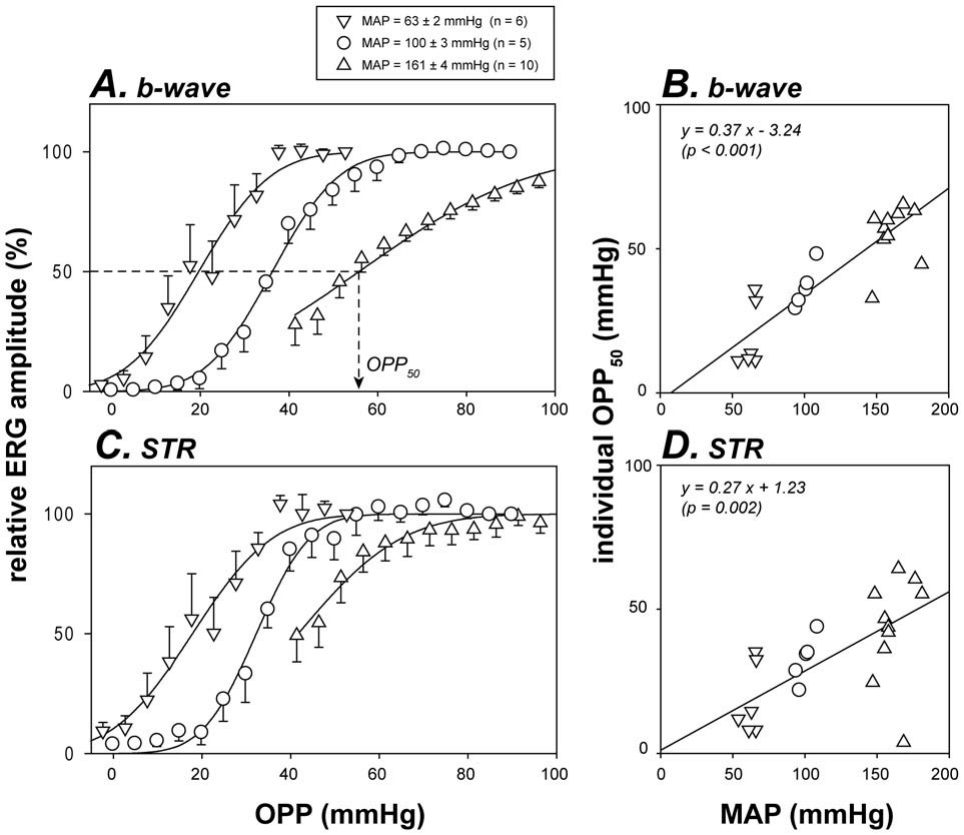
Relationship between retinal function and ocular perfusion pressure. Retinal function (mean & SEM) is plotted as a function of OPP for various BP groups. Data for b-wave (**A**) and STR (**C**) are reproduced from Figures 3B and 3E respectively. Curves: cumulative normal function as per Figure 3 (Equation 1). Arrow: definition of OPP50 as the OPP for 50% functional deficit. Relationship between individual OPP50 and MAP was correlated with Deming regression (lines in **B & D**).

The susceptibility of retinal function to elevated IOP was quantified using a cumulative normal function (Equation 1, curves in Figures 3B and 3E), which returns the IOP for 50% dysfunction (IOP_50_, arrow in Figure 3B). As shown in Figures 3C and 3F, individual IOP_50_ for both b-wave and STR amplitude increased proportionately with MAP. Deming regressions showed a significant correlation with a slope of less than 1 (p<0.001 for both Figures 3C and 3F). A positive correlation between MAP and IOP_50_ indicates that hypertension increases the ability of neurons to resist IOP insult. However, a slope of less than unity suggests that MAP elevation does not *fully* compensate for the retinal dysfunction induced by the same amount of IOP elevation. This issue is better illustrated when retinal function is plotted against OPP (Figure 4).

If OPP were the sole determinant of retinal function then relative function for all three blood pressure groups should overlie each other when plotted against OPP. In contrast, the OPP-response functions clearly did not overlie each other. Moreover, when modeled with a cumulative normal function (Equation 1, curves in Figure 4), the OPP_50_ (the OPP for 50% functional loss) should be the same for all animals regardless of blood pressure. However, this was not the case. The presence of a significant positive correlation between OPP_50_ and MAP (Figures 4B and 4D, Deming regression, all p<0.05) shows that function in the hypertensive group was not as well protected as one might expect from the OPP improvement, whereas for the low BP group susceptibility was not as bad as anticipated from the change in OPP.

### Influence of blood pressure on vascular susceptibility to IOP

**Figure 5:**
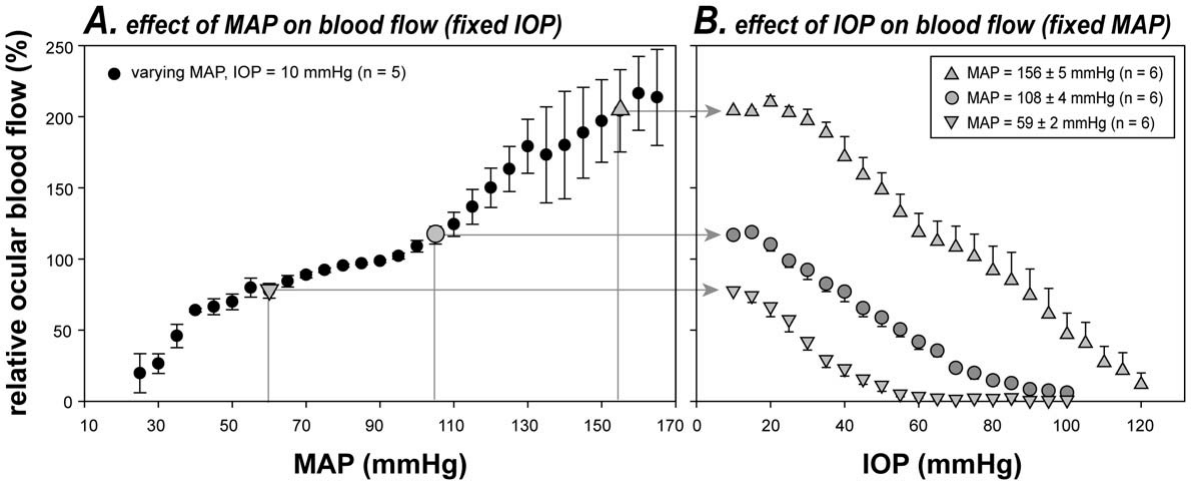
Effect of BP on the susceptibility of ocular blood flow to IOP elevation. Relative ocular blood flow (± SEM) during MAP (A, fixed IOP) and IOP manipulation (B, fixed MAP). Data in A shows characteristics of blood flow autoregulation as evident by a relative plateau at intermediate MAPs. As LDF does not measure absolute flow, the autoregulation curve in A is used to set the starting blood flow level for the three blood pressure groups (arrows in B). Thus all data are effectively normalized to a “normal” baseline condition (in A, IOP 10 mmHg, MAP 93±3 mmHg, n = 5).

To consider whether the retinal dysfunction found during OPP changes (Figures 2 to [Fig pone-0031104-g003] 4) was mediated by blood flow, Figure 5 compares the IOP-flow relationships for high, moderate and low BP groups. Unlike the functional study (Figure 3) where baseline ERG in all groups could be normalized to 100%, baseline ocular blood flow varies with BP and therefore cannot be set to the same level. Figure 5A shows that baseline ocular blood flow increased with MAP elevation, except at an intermediate level where there was a plateau, consistent with autoregulation. This curve provides a means to determine the starting point for blood flow in the three different BP groups. At baseline IOP (10 mmHg), the average MAP for the low, moderate and high BP groups was 59, 108 and 156 mmHg, which corresponds to a relative blood flow of 78%, 116% and 204%, respectively (arrows in Figure 5). Here 100% represents the normal pre-manipulation condition (IOP 10 mmHg, MAP 93±3 mmHg). Figure 5B shows that relative ocular blood flow when plotted as a function of IOP (Figure 5B) was shifted to higher IOPs in animals with acute high BP, and to lower IOPs in animals with low BP (interaction, p<0.001, two-way RM ANOVA).

### Comparing functional and blood flow response to OPP

**Figure 6:**
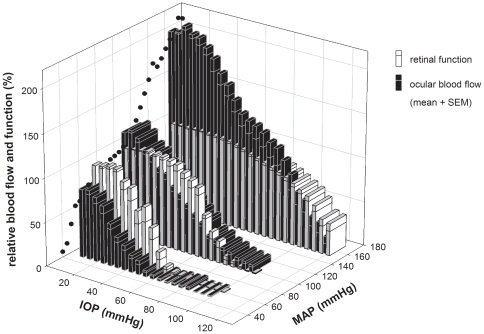
Comparison of relative retinal function and ocular blood flow during IOP elevation. Data for animals with acute low, moderate, and high BP are re-plotted here. Black circles: blood flow autoregulation curve at baseline IOP (reproduced from Figure 5A). Function (unfilled bars, b-wave amplitude) is normalized to baseline. Starting blood flow (filled bars) is adjusted based on the autoregulation curve. Bars represent mean + SEM.

As summarized in Figure 6, both retinal function and blood flow were progressively reduced with lower levels of OPP, achieved with high IOP or low MAP. However, retinal function and ocular blood flow clearly did not decline at the same rate. At low and moderate MAP, retinal function remained relatively preserved despite substantial IOP-induced blood flow deficiency, suggesting that a “functional reserve” protects against relative ischemia. However, the relationship between function and blood flow was reversed in the high BP group. Retinal function begins to decline when IOP was at 60 mmHg, but blood flow was still well above baseline. This outcome suggests that retinal function has a ceiling and does not improve with hyper-perfusion.

## Discussion

### Effect of OPP on retinal function

A key finding of this study was that OPP (determined by BP and IOP) substantially influences retinal function. In animals with low BP, milder IOP elevations were needed to compromise retinal function compared with controls. In contrast, animals with an acute increase in BP endured a greater IOP challenge before retinal dysfunction occurred (Figure 3). These results confirm previous studies which have found that bipolar cell function, measured with the ERG b-wave [24], and ganglion cell function, measured with axonal impulse conduction [23] or pattern ERG [24], were affected by the balance between BP and IOP. Therefore, OPP is a more important determinant of neuronal function, than IOP alone.

In addition, the current study extends previous work [23], [24] to show that retinal susceptibility to a common IOP challenge (quantified as IOP_50_) is linearly related to BP (Figures 3C and 3F). However, OPP was not the only determinant of retinal function. Had IOP elevation and BP reduction produced the same effect, the relationship between IOP_50_ and MAP (Figures 3C and 3F) would have returned a unity line. In fact, a slope of less than one showed that as BP increased, the increment in IOP_50_ was less than expected based solely on OPP. In other words, MAP elevation cannot fully compensate for the retinal dysfunction induced by the same amount of IOP elevation. This was somewhat counterintuitive given that OPP, by definition, contains the same contribution from MAP and IOP (*i.e.* OPP = MAP−IOP).

That IOP is more important than BP in determining retinal function was further illustrated in Figure 4, wherein retinal function plotted against OPP did not collapse to a single curve. That is, for a given OPP, the higher IOP elevation induced greater retinal dysfunction. This is possibly because BP modification influences vascular supply only, whereas an IOP elevation affects the vascular supply via a reduction in OPP, and produces mechanical stress on retinal neurons that is OPP independent. Although previous studies [23], [24] have also assessed retinal function during IOP and BP manipulation, those studies did not independently compare the influence of BP and IOP. To our knowledge, this is the first study to show that IOP is more important than BP in determining retinal function for a given OPP.

### Comparing retinal function and ocular blood flow

Measurement of ocular blood flow by laser Doppler indicates that the IOP-flow relationship was rightward shifted with increasing BP (Figure 5B), indicating that BP modifies susceptibility to IOP challenge. This finding agrees with previous studies of ocular blood flow as recently reviewed by Schmidl and colleagues [44]. In particular, Kiel and van Heuven [29] showed in rabbit eyes that it took more IOP elevation to reduce choroidal blood flow as MAP increased (20 to 80 mmHg). The current study extends their observations to include higher MAPs (108 and 156 mmHg). More recently, Liang and colleagues [45] also showed that IOP-induced ischemia at monkey optic nerve head is exacerbated by systemic hypotension (MAP 56 mmHg).

As it is technically difficult to monitor retinal function and blood flow simultaneously whilst concurrently manipulating IOP and BP, we adopt an alternate approach. We measured these outcomes in separate groups of animals under closely matched experimental conditions (same IOP and similar MAP modifications, Figure 1). Figure 6 showed that BP modifies vascular (filled bars) and functional (unfilled bars) susceptibility to IOP elevation in a similar way. This suggests that the protection afforded by acute high BP on retinal function is in part mediated by improved blood flow. That retinal function was relatively preserved despite substantial blood flow reduction (Figure 6) provides evidence for a “functional reserve” [22]. One mechanism that could underlie this functional reserve is a capacity to increase oxygen extraction from residual arterial blood during mild ischemia, which helps to maintain tissue oxygenation [22]. With low perfusion pressure it would be predicted that erythrocyte transit would be slower and oxygen extraction may be increased.

The current study sought to understand basic retinal physiology in terms of functional and hemodynamic response to OPP stress, therefore only acute IOP and BP manipulations were employed. Interpretation of our findings in the context of chronic diseases such as glaucoma and essential hypertension requires caution. In future studies, it will be of interest to examine the effect of chronic hypertension on the susceptibility to IOP challenge.

### Summary

In summary, the susceptibility of retinal function to IOP challenge can be partially ameliorated by acute high BP, and exacerbated by low BP. The mechanism behind this change can be partly attributed to alterations in OPP and its effects on ocular blood flow. We find that retinal function can be normal even when blood flow is partially reduced. This may reflect the presence of an additional compensatory mechanism, possibly related to the capacity to regulate oxygen extraction. Interestingly, IOP elevation produces more dysfunction than does BP reduction of the same magnitude. This difference is likely to reflect the potential for IOP to produce both vascular insufficiency and mechanical stress on retinal neurons.

## Supporting Information

Figure S1 **Comparison of IOP elevation induced by anterior and posterior chamber cannulation.** Both anterior (A) and vitreal chamber (B) show a strong and similar linear relationship with IOP elevation induced via anterior chamber cannulation.
(TIF)

Figure S2 **Effect of SNP, saline and AngII on retinal function.** Relative retinal function remained stable in the sham control eye (IOP = 10 mmHg) during continuous infusion of SNP (A & D), saline (B & E) or AngII (C & F) to sustain low, moderate or high blood pressure. Time “0” represents the beginning of stepwise IOP elevation in the fellow eye. A, B & C: relative b-wave amplitude; D, E & F: relative STR amplitude; Shaded area: 95% confidence intervals for b-wave and STR amplitudes at time “0”.
(TIF)

Figure S3 **Effect of 100% O_2_ breathing on ocular blood flow. Backscatter (A), MAP (B) and ocular blood flow (C) were measured before, during and after100% oxygen breathing.** Error bars: SEM; n = 6. Shaded area: duration (2 minutes) of 100% oxygen administration. Dashed line: baseline blood flow (100%).
(TIF)
