# Supplementary figures and images for: Blood Pressure Modifies Retinal Susceptibility to Intraocular Pressure Elevation
Source: PLoS One. 2012 Feb 16;7(2):e31104. doi: 10.1371/journal.pone.0031104 (PMC3281054; doi:10.1371/journal.pone.0031104)

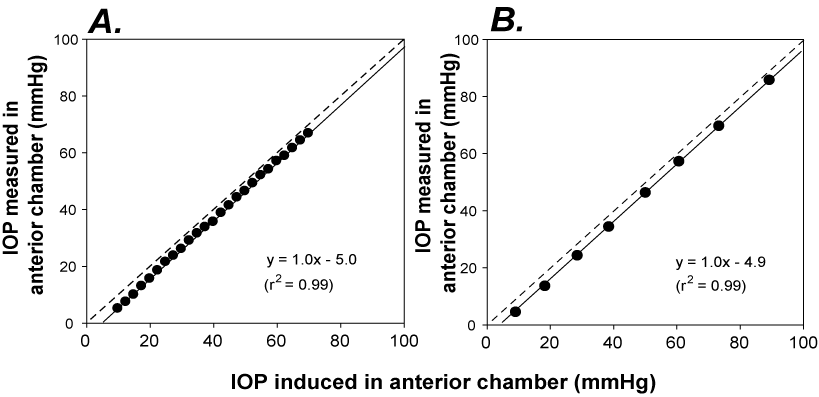

Supplement: Figure S1 — Comparison of IOP elevation induced by anterior and posterior chamber cannulation. Both anterior (A) and vitreal chamber (B) show a strong and similar linear relationship with IOP elevation induced via anterior chamber cannulation. (TIF) [file pone.0031104.s001.tif]

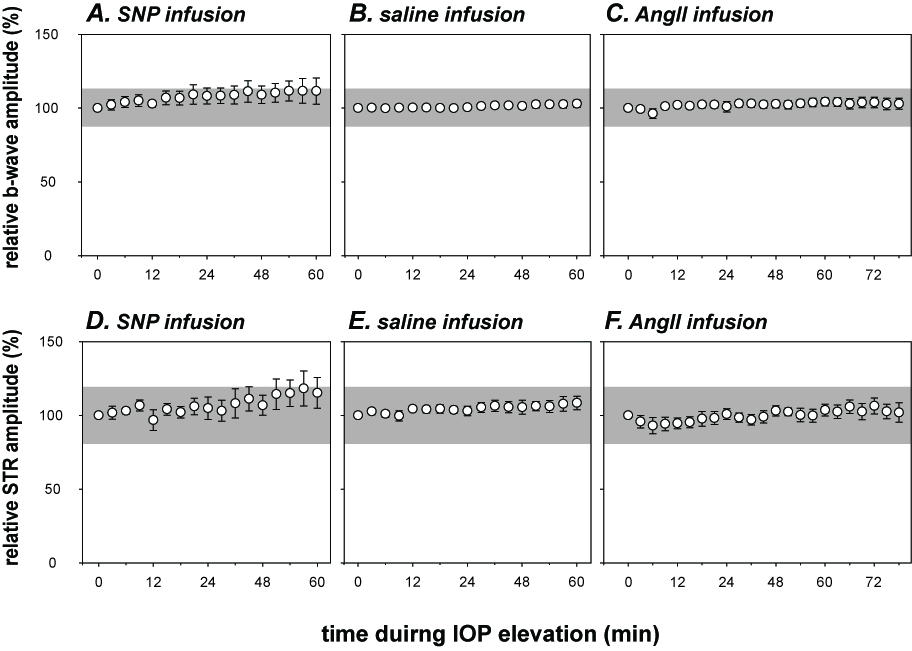

Supplement: Figure S2 — Effect of SNP, saline and AngII on retinal function. Relative retinal function remained stable in the sham control eye (IOP = 10 mmHg) during continuous infusion of SNP (A & D), saline (B & E) or AngII (C & F) to sustain low, moderate or high blood pressure. Time “0” represents the beginning of stepwise IOP elevation in the fellow eye. A, B & C: relative b-wave amplitude; D, E & F: relative STR amplitude; Shaded area: 95% confidence intervals for b-wave and STR amplitudes at time “0”. (TIF) [file pone.0031104.s002.tif]

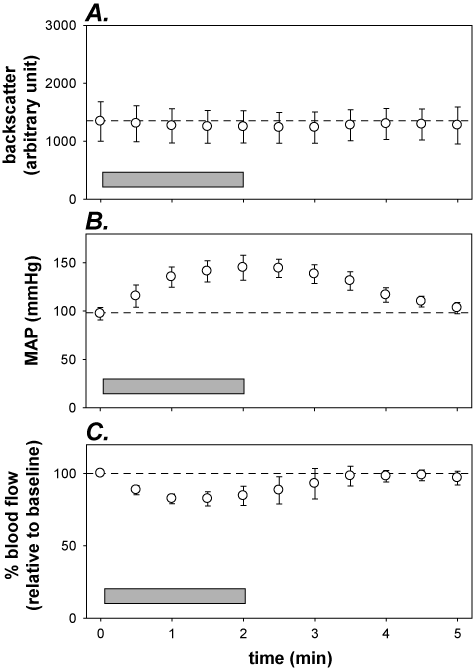

Supplement: Figure S3 — Effect of 100% O2 breathing on ocular blood flow. Backscatter (A), MAP (B) and ocular blood flow (C) were measured before, during and after100% oxygen breathing. Error bars: SEM; n = 6. Shaded area: duration (2 minutes) of 100% oxygen administration. Dashed line: baseline blood flow (100%). (TIF) [file pone.0031104.s003.tif]
